# High-yield isolation of extracellular vesicles using aqueous two-phase system

**DOI:** 10.1038/srep13103

**Published:** 2015-08-14

**Authors:** Hyunwoo Shin, Chungmin Han, Joseph M. Labuz, Jiyoon Kim, Jongmin Kim, Siwoo Cho, Yong Song Gho, Shuichi Takayama, Jaesung Park

**Affiliations:** 1 Department of Mechanical Engineering, POSTECH, 77 Cheongam-Ro, Nam-Gu, Pohang, Gyeongbuk, 790-784, Republic of Korea; 2 School of Interdisciplinary Bioscience and Bioengineering, POSTECH, 77 Cheongam-Ro, Nam-Gu, Pohang, Gyeongbuk, 790-784, Republic of Korea; 3 Department of Life Sciences, POSTECH, 77 Cheongam-Ro, Nam-Gu, Pohang, Gyeongbuk, 790-784, Republic of Korea; 4 Department of Biomedical Engineering, College of Engineering, Biointerfaces Institute, University of Michigan, 2800 Plymouth Rd, Ann Arbor, USA; 5 Macromolecular Science and Engineering Center, College of Engineering, Biointerfaces Institute, University of Michigan, 2800 Plymouth Rd, Ann Arbor, USA

## Abstract

Extracellular vesicles (EVs) such as exosomes and microvesicles released from cells are potential biomarkers for blood-based diagnostic applications. To exploit EVs as diagnostic biomarkers, an effective pre-analytical process is necessary. However, recent studies performed with blood-borne EVs have been hindered by the lack of effective purification strategies. In this study, an efficient EV isolation method was developed by using polyethylene glycol/dextran aqueous two phase system (ATPS). This method provides high EV recovery efficiency (~70%) in a short time (~15 min). Consequently, it can significantly increase the diagnostic applicability of EVs.

Extracellular vesicles (EVs) such as exosomes and microvesicles are small membrane vesicles (50–1000 nm) that exhibit characteristics of the cells and tissues from which they are derived^1^,^2^,^3^,^4^. However, using EVs directly for diagnosis is challenging owing to the limitations of current isolation methods that provide a low yield and/or a low purity of EVs. Therefore, to improve the diagnostic utility of EV, an isolation method enriches EVs at a high concentration and purity is required.

Currently, EVs are isolated by a variety of methods such as ultra-centrifugation (U/C), size exclusion, immunoaffinity, microfluidics and polymeric precipitation^5^. U/C is currently the favoured method to isolate EVs due to its reliability^6^,^7^. However, U/C is a lengthy and costly process, yet isolates only a small portion of the EVs; for example, the estimated isolation yield of U/C from culture media is 5–25%^8^. Size exclusions (e.g., filters or chromatography) are often used in conjunction with U/C or other techniques, but have low yield because they lose a large amount of EVs because they due to adhesion of EVs to filters^5^,^6^,^9^,^10^,^11^. Immunoaffinity isolation methods use antibodies that attach to desired EV populations or unwanted EV populations^6^,^11^,^12^,^13^. However, this method is not appropriate for practical isolation because isolation requires physical contact between EVs and capturing molecules, which is costly^14^,^15^,^16^,^17^. Recently, the feasibility of using microfluidic chips isolate EVs based on immunoaffinity or size exclusion has been assessed^9^,^18^. Although isolating EVs using microfluidics can exploit the platforms developed for EV isolation, the methods have low throughput and are therefore not suitable for pre-analytical processes. In summary, all current EV isolation achieve low yields, and this low efficiency may misrepresent the true EV population in blood.

Polymeric methods reduce EV solubility and drive precipitation by dissolving polymers. These methods achieve a higher yield than U/C and may therefore be an alternative to it^19^,^20^. However, they typically require long incubation times to precipitate EVs, and cannot appreciably purify EVs from a protein mixture^19^. This method decreases the solubility of almost all particles such as EVs and proteins in solution^21^. As a result, proteins are co-precipitated with EVs, and contaminate the isolates. According to previous studies of EV isolation, both low yield and impurity contamination are problematic for further applications, and an alternative approach is demanded to achieve higher yield with better purity.

Aqueous two phase systems (ATPSs) can separate particles because different kinds of particles are effectively partitioned to different phases in a short time. ATPSs have been used to separate cells that have different membrane surface properties, to separate proteins, to extract testosterone and epitestosterone for doping tests, and to improve the sensitivity of polymerase chain reaction (PCR) detection by extracting PCR inhibitors^22^,^23^,^24^,^25^,^26^.

In this paper, we use a polyethylene glycol (PEG) / dextran (DEX) ATPS to isolate EVs from a mixture of EVs and proteins. EVs were isolated in the DEX phase with efficiency of ~70% at the optimal polymer concentration. This ATPS isolates EVs effectively, and has obvious applications in diagnostic applications of EVs.

## Results and Discussion

### Principle of particle separation using ATPS

If the particles diffuse freely, partition in ATPS can be represented using the Boltzmann equation


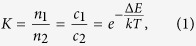


where *K* is a partition coefficient, *n*_1_ and *n*_2_ are the numbers of particles in phases 1 and 2 respectively, *c*_1_ and *c*_2_ are the concentrations [mol∙L^−1^] of particles phase 1 and 2, Δ*E* [J] is energy needed to move the particle to another state, *k *= 1.381 × 10^−27^ J∙K^−1^ is Boltzmann’s constant, and *T* [K] is absolute temperature.

Δ*E* between states is determined by the surface free energy of particle because particle partition in ATPS is mainly affected by a surface between particle and solution. Therefore, *K* can be calculated from the difference in surface free energies of a particle in the two phases. Several factors affect this difference, including (1) Van Der Walls interactions or dispersion forces, (2) hydrogen bonding, (3) hydration, (4) hydrophobic interaction, (5) electrostatic interaction, and (6) polymer and ion binding or repulsion^27^. These factors are coupled, so their individual contributions to the free energy of a particle cannot be separated easily.

To calculate the free energy of a particle, Flory-Huggins theory^27^ partitions change Δ*G* [J] in free energy into change Δ*H* [J] in enthalpy and change *T*Δ*S* [K∙J∙K^−1^] in entropy:


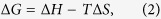


We used Flory-Huggins theory to obtain ΔG.

According to the equation derived from Flory-Huggins theory, particle separation in ATPS is expressed as^28^.


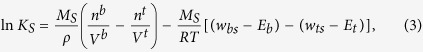


where subscripts *b* and *t* represent fractions in the bottom and top, respectively; partition coefficient *K*_*s *_= *s*_*b*_*/*s_t_, where s_i_ (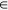 *B*, *T*) is the volume fraction of the molecule in *i*, *M*_*s*_ [g] is the mass of the solute, *ρ* [g∙m^−3^] is the number of lattice sites per unit volume, *n*^*i*^ are the numbers of molecules, and *V*^*i*^ [m^3^] are volumes, *R *≈ 8.83 J∙K∙mol^−1^ is the gas constant, *T* is absolute temperature, *w*_*ts*_ [J] and *w*_*bs*_ [J] are respectively the energy of interaction between the particle’s lattice site and the average lattice site of the top phase and the average lattice site bottom phase, and *E*_*i*_ [J] is the binding energy between average lattice sites of the bottom and top phases. The first term on the right-hand side of Equation (3) is the entropic term derived from Flory-Huggins theory by ignoring the enthalpic contribution; this term shows that a particle’s partition in an ATPS is related to the number density of each phase. This term predicts that particles will move to the phase that has the highest number density. The second term in Equation (3) is the enthalpic term derived from the same theory by ignoring the entropic contribution, and explains how molecular interactions such as hydrophobic, hydrophilic and electrostatic affect the partitioning of particles. For example, small 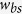 means that particles are strongly attracted by the bottom phase and will therefore move to it. The self-energies *E*_*t*_ and *E*_*b*_ are determined by the amount of energy required for particles to break existing interactions and enter phases; the energies are proportional to the concentrations of phase-forming polymers. Further details of the equations are provided in supplementary information (Supplementary Equation S1).

### EV preparation and validation

In order to determine an appropriate ATPS composition, sufficient amount of EVs are required for numerous iterations to determine the system composition. In addition, recent studies discovered that some proteins highly expressed in tumor interstitial fluid (alpha-enolase, L-lactate dehydrogenase, glutathione S-transferase P) were also found to be secreted through tumor-derived exosomes^29^,^30^,^31^,^32^. In this sense, tumor interstitial fluid provides a good source of EVs, which contains large amount of EVs from many different tissues. We isolated EVs from tumor interstitial fluid using conventional U/C EV preparation method.

**Figure 1:**
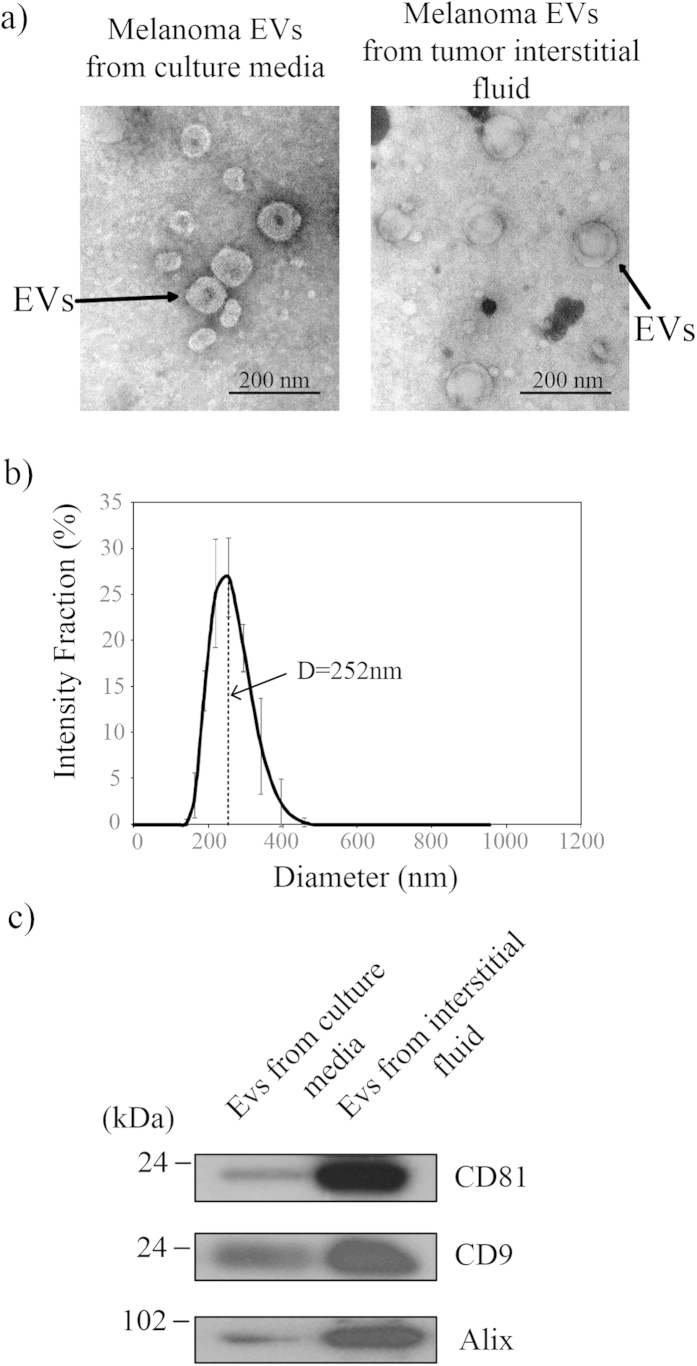
Melanoma EVs were isolated by ultra-centrifugation from culture media and tumor interstitial fluid. (**a**) Morphological characteristic of the both EVs were analyzed using TEM images. (**b**) Size distribution of interstitial fluid EVs were measured using DLS. (**c**) Existence of EV surface marker was analyzed by CD81, CD9, and Alix western blot with 2 μg of proteins from U/C culture media and tumor interstitial fluid. The gels have been run under the same experimental conditions.

To determine whether EVs isolated from tumor interstitial fluid are similar morphologically to those isolated from conventional cell-culture medium, we visualized the both EVs using TEM (Fig. 1a). EVs from both tumor interstitial fluid and cell cultured medium were cup-shaped. Widely-used EV surface markers CD81, CD9, and Alix in EVs from both sources were analyzed using western blotting (Fig. 1b); bright bands at the expected sizes of CD81, CD9, and Alix were obtained from both of the EV samples. Particularly, the CD81 and Alix signals from the tumor interstitial fluid EV sample were much brighter than those from isolated from conventional cell-culture medium; this difference implies that these two markers are enriched in tumor interstitial fluid EV. EVs from tumor interstitial fluid were used as the source of quantity-defined EV-protein mixture for further isolation experiments because interstitial EVs provide not only enough amounts but also heterogeneous populations of EVs. Addtionally, the size distribution of interstitial fluid EVs was identified by using dynamic light scattering (Fig. 1c). The size of interstitial fluid EVs ranged 140 nm to 460 nm, and average size was 252 nm. EVs from tumor interstitial fluid were used as the source of quantity-defined EV-protein mixture for further isolation experiments.

### Optimization of EV isolation by controlling polymer composition

**Figure 2:**
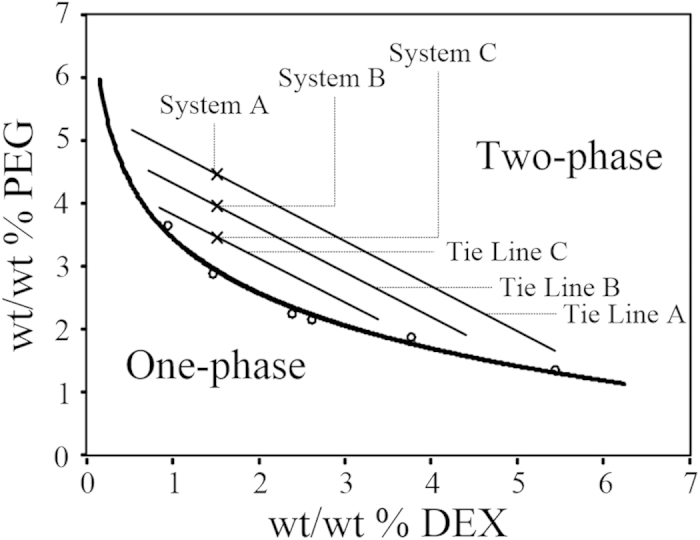
Phase diagram of PEG/DEX ATPS for DEX and PEG in PBS solution. Aqueous two-phase systems can only form at combinations above the curve. Experiments were performed with the three systems shown in the figure which can represent all systems on the same tie-lines.

To determine the optimal polymer composition of ATPS for EV isolation, three different PEG/DEX compositions along different tie-lines were investigated. PEG and DEX concentrations were selected to be above the phase transition line in the phase diagram (Fig. 2)^27^. Systems that contain more than 5% wt/wt of either polymer were excluded because these polymer concentrations require long dissolution time and generate high interfacial tension. The partition behavior analyzed in the three selected systems (A, B and C) can represent every other system on the same tie-line. One system point in the two-phase diagram should encounter one tie-line that passes it (Fig. 2). The EV partitioning behaviors of every different system points on the same tie-line are expected to be the same because every system on a given tie-line has the same final polymeric composition and the same interfacial tension; thus it has the same partition coefficient^27^. Partition coefficients of the systems are defined as the ratio of EV or protein concentration in the phases post-isolation: *k *= (concentration in DEX phase/ concentration in PEG phase).

**Figure 3:**
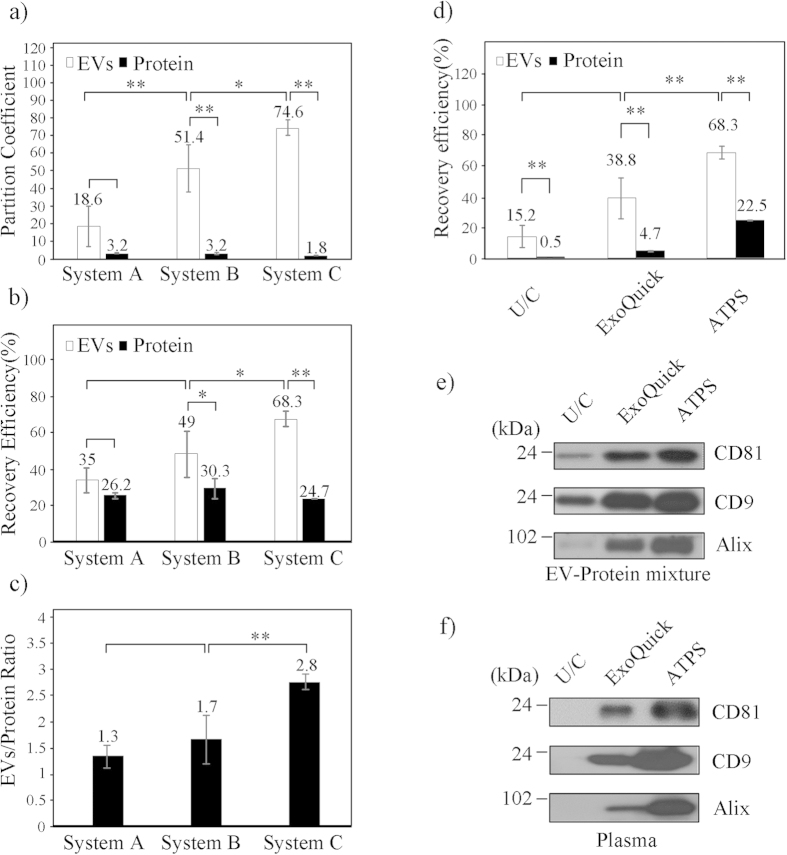
Characterization and identification of ATPS comparing to conventional methods. (**a**) Partition coefficient of EVs and proteins. A large partition coefficient means that the EVs or proteins are highly concentrated in DEX-rich phase. The partition coefficient of EVs was calculated by measuring RNA concentrations in post-isolation PEG-rich and DEX-rich phases, and the partition coefficient of protein was calculated by measuring protein concentrations in post-isolation PEG-rich and DEX-rich phases. (**b**) Recovery efficiency of EVs and proteins. A high recovery efficiency means that a large proportion of EVs or proteins moved to the DEX-rich phase. Recovery efficiency of EVs is calculated by dividing the RNA amount of post-isolation DEX-rich phase by the RNA amount of the original EV-protein mixture; recovery efficiency of protein is calculated in the same manner, using protein amount instead of RNA amount. (**c**) The efficiency ratio of EV with respect to protein was calculated as a parameter for EV purity evaluation. The results were analyzed by ANOVA with Tukey’s test. Significant differences in selected pairs are noted as *p < 0.05, **p < 0.01. (**d**) Recovery efficiency comparison of U/C, ExoQuick^®^, and ATPS. Optimized ATPS method showed almost 4 times and 2 times higher EV recovery efficiency compared to U/C and ExoQuick^®^, respectively. (**e**) CD81, CD9, and Alix western blot performed with EVs isolated by U/C, ExoQuick^®^, and ATPS methods from EV-protein mixture. Total isolated EV samples of 2 μl from U/C, ExoQuick^®^, and ATPS method were used. Signals of markers from EVs obtained using ATPS were significantly stronger those obtained using U/C or ExoQuick^®^. The EV concentration obtained using U/C was too low, so Alix could not be detected. (**f**) CD81, CD9, and Alix western blot performed with EVs isolated by U/C, ExoQuick^®^, and ATPS method from mouse plasma. The western result from mouse plasma shows similar tendency as EV-protein mixture. The gels have been run under the same experimental conditions. The results were analyzed using ANOVA and Tukey’s test. Significant differences in selected pairs are noted as *p < 0.05, **p < 0.01.

EVs were quantified using RNA amount, and proteins were quantified using a Bradford protein assay^5^. Partition coefficient analysis of three different PEG/DEX ATPSs showed that EVs have significantly higher (~18 times higher in system A, ~51 times higher in system B and ~74 times higher in system C) affinity to the DEX-rich phase than to the PEG-rich phase in all three systems, whereas proteins had comparatively low affinity to DEX-rich phase in all three systems (Fig. 3a). The partition coefficients of EVs and proteins were significantly different in both the system B and C (p < 0.01, Tukey’s test) but not in system A (p > 0.1, Tukey’s test). Particularly, ATPS with the lowest polymer concentration (system C) showed the highest EV partition coefficient and lowest protein partition coefficient simultaneously.

The partition coefficient analysis gave only the information about concentration ratio, not about amounts, so the quantity of partitioned EVs and proteins was calculated by multiplying the corresponding volumes of each post-isolation samples. Therefore, we calculated recovery efficiency of EVs and proteins, which is defined as percentage of DEX-rich phase partitioned EVs and proteins from the EV-protein mixture (Recovery efficiency: *E *= Partitioned amount in DEX-phase/Initial amount in EV-protein mixture) (Fig. 3b). Systems A and B isolated less than half of the EVs from the EV-protein mixture in DEX-rich phases (system A: ~ 35%; system B: ~ 49%), but system C isolated ~68% of the EVs. Protein-recovery efficiency was very similar in the three systems (p > 0.8, Tukey’s test).

This difference in partition coefficient and recovery efficiency between the systems might be caused by the difference in interfacial tension. The interfacial tension can be inferred from the calculated amount of trapped EVs and proteins at the phase interfaces of the systems. The ATPS with the lowest polymer concentration (system C), might also have the lowest interfacial tension, and thus the fewest EVs were trapped at the interface of system C among the all three systems: by calculations, ~26% of EVs were trapped at the interface in system C, but ~40% and ~44% of EVs were trapped at the interfaces in system A and B, respectively. The amount of proteins trapped at the interface was <2% in all cases; this result is in agreement with previously-described surface free energy theory, which explains the effect of interfacial tension on partition of different-sized particles in ATPS: partition of proteins, which are generally much smaller than EVs, was mostly unaffected by interfacial tension difference^22^. Because protein impurities in isolated EV samples are problematic for applications that use EVs, we introduce the EV/protein recovery efficiency ratio as a simple parameter to evaluate the relative purity of isolated EVs. Again, system C had the smallest amount of protein impurity (Fig. 3c).

To summarize, the results partition coefficient and recovery efficiency analyses indicate that the lowest polymer concentration tested gave the best EV isolation. Therefore, we selected system C for further analyses. A system with lower polymer concentration than system C, which was expected to show better isolation efficiency, generated unstable phase separation because the concentration of the system was too close to the point at which a two-phase system turns into a one-phase system.

### Comparison between ATPS and other methods

#### Detection of Protein marker from interstitial and plasma EVs isolated using ATPS

To evaluate EV-separation efficiency of ATPS, the EV recovery efficiencies of U/C, ExoQuick^®^ and ATPS methods were compared. The ATPS isolation method optimized by adjusting the polymers’ concentration recovered ~70% EVs from the EV-protein mixtures, whereas U/C recovered only <16% and and ExoQuick^®^ recovered only 40% (Fig. 3d). EV recovery efficiency of ATPS method was at least four times larger than that of U/C (p < 0.01, Tukey’s test). An earlier publication also reported that EV recovery efficiency of a polymeric method was nearly twice that of U/C^19^.

The advantage of this high recovery efficiency of ATPS method was confirmed by comparing the signal strength of western blot analysis of EV-specific markers CD81, CD9, and Alix. Among the three methods, ATPS yielded the brightest bands of these markers (Fig. 3e); this result corresponds to the comparative EV recovery efficiencies (Fig. 3d). In addition, the three markers show similar western blot results, which means that ATPS successfully isolated heterogeneous EVs. EV markers were difficult to identify in samples purified using U/C because this method has very low EV isolation efficiency (~10%).

To confirm the ability of ATPS to isolate EVs from body fluid, EVs isolated from mouse plasma were also analyzed using the same western blot experiment. EVs were isolated successfully despite the large amount of protein in plasma (Fig. 3f). ATPS yielded the strongest CD81, CD9, and Alix bands. This results supported that heterogeneity of EVs were successfully isolated by ATPS from blood plasma.

#### Morphology and RNA content of EVs

**Figure 4:**
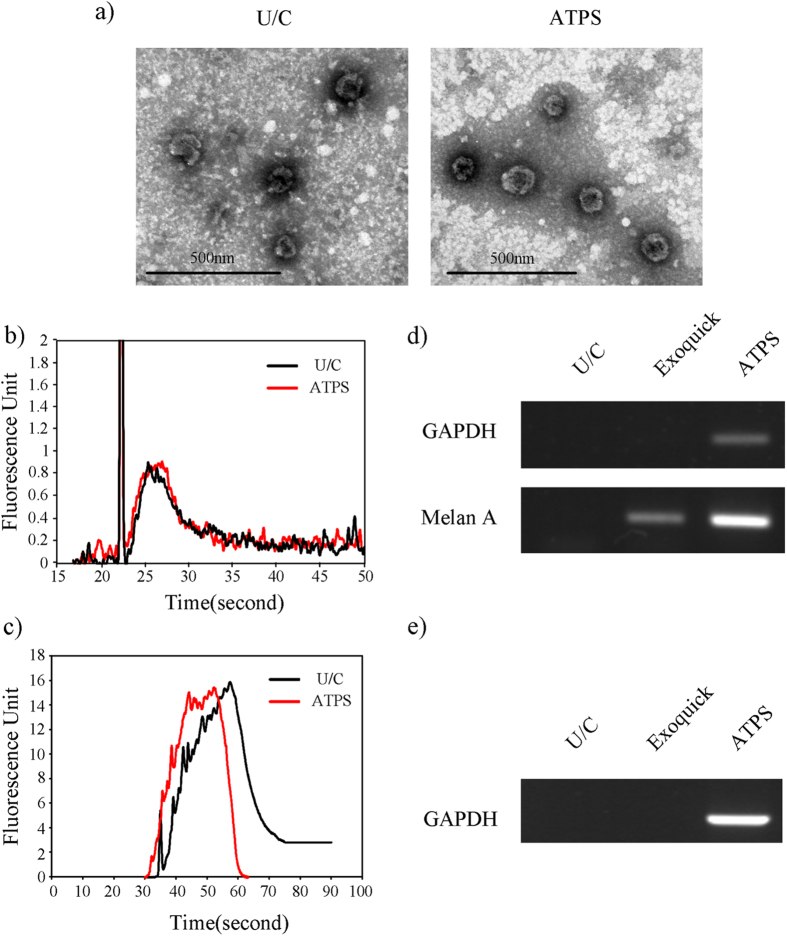
Identification of EV quality and utility of ATPS for RT-PCR (**a**) TEM picture did not show any morphological differences between EVs isolated using ATPS and U/C methods. (**b**) Total RNA profile comparison between U/C and ATPS. Qualitative RNA analysis showed that the EV RNA profiles from the ATPS and U/C methods almost overlap. Time axis represents the size (nt) of the RNA; fluorescence intensity (y-axis) of the graph represents the relative amount of RNAs at corresponding sizes. These two results imply that ATPS isolation method did not damage the EVs. (**c**) Small RNA profile comparison between U/C and ATPS. Small RNA analysis showed slightly different small RNA profiles between ATPS and U/C. (**d**) Reverse transcription-PCR was performed using 4.5 μl of previously prepared EV RNA samples from the original EV-protein mixture and the ATPS and U/C methods. GAPDH was detected only from RNAs obtained using he ATPS method. Melanoma marker Melan-A was detected from RNAs obtained using ATPS or ExoQuick method, ATPS method showed significantly brighter bands for the given volumes. U/C sample yielded an EV concentration that was too low to be detected by RT-PCR for the given volumes. (**e**) Reverse transcription-PCR was also performed in blood plasma. GAPDH was not detected from EVs obtained using U/C or ExoQuick^®^ blood plasma, but bright band of GAPDH was detected from EVs obtained using ATPS.

In addition to western blot analysis, the morphology and RNA content of the EVs were examined to determine their quality. In TEM images, EVs isolated using ATPS did not differ morphologically from EVs obtained using U/C (Fig. 4a). For analysis of RNA contents, the same amount of RNAs isolated from EVs isolated using ATPS and using U/C were compared using a bio-analyzer (Fig. 4b). In both, the main peak appeared near 28 s, and the areas under the curves were similar, so the EVs isolated using the two methods contain similar amounts of RNA. The peak appeared near 22 s shows just standard marker. The overall profiles of RNAs prepared from the both sets of EVs overlapped almost completely; this similarity implies that the ATPS EV isolation method does not have adverse effects on isolated EVs’ total RNA contents. However, small RNA profile differed slightly between ATPS and U/C method (Fig. 4c). Especially, the profile of small RNA in ATPS is slightly shifted to left comparing to profile obtained using U/C. The shifting effect might be induced by dextran that remained after RNA isolation because the dextran increases the viscosity of the samples, and this increase affects the detection of small RNA. Concentration of dextran that was used for ATPS is low, so the shifting effect is minor. Additionally, a high concentration of polymers may inhibit PCR because extremely high viscosity prevents chemical reaction. The polymer concentration of ATPS is low (1.5%), so the viscosity did not inhibit PCR. The experiment is explained in detail in the following section.

#### Detection of RNA marker from interstitial and plasma EVs isolated using ATPS

To demonstrate the diagnostic applicability of the ATPS isolation system, we conducted reverse transcription PCR for detection of melanoma-related cancer markers in RNA samples prepared from EVs isolated using ATPS. The presence of melanoma marker (Melan A) in EVs was clearly confirmed in the sample isolated using ATPS; and the PCR band that sample was significantly brighter than the bands obtained using U/C or ExoQuick^®^ (Fig. 4c). This result corresponds well with the results of the western blot analysis in the previous section. To confirm the ability of ATPS to isolate EVs from body fluid, EVs isolated from mouse plasma were also analyzed using the same reverse transcription PCR experiment; results (Fig. 4d) were similar to those in Fig. 4c. Although the amount of protein amount in blood plasma is large, GAPDH was successfully detected from the EV isolated from plasma by ATPS. Moreover, GAPDH was detected only in EVs isolated by ATPS because of its high EV recovery efficiency.

In summary, although 22.5% of the protein still remained via ATPS from plasma protein, this protein impurity did not interfere with further western blot or PCR analyses of EVs. In the case of western blot, PSMA, prostate specific membrane antigen, is successfully detected from not pre-cleaned blood serum in previous research^33^. This result supports that the amount of protein remaining is not large enough to hinder a feasibility of the western blot. In the case of PCR, most of plasma protein was eliminated in RNA extraction step, and PCR detection was affected much by amount of target RNA. Therefore, the recovery efficiency of EVs is more strongly affect the assays than a purity of isolated EVs.

## Conclusion

We developed a high-yield EV-isolation method that uses a concentration-adjusted PEG/DEX aqueous two-phase system, and that does not require long incubation times or specialized laboratory equipment. Biased affinity of EVs to the DEX-rich phases results in ~4 times higher recovery efficiency than achieved using U/C. The proposed method would allow easy extraction of EVs from biological samples. The ease of the isolation procedure might contribute to popularize applications of EVs. We confirmed the diagnostic applicability of our isolation method by performing western blot of EV-specific markers, and RT-PCR detection of EV in blood plasma. This wide compatibility of the isolation method will simplify the task of finding EV biomarkers for disease diagnosis and prognosis.

## Materials and Methods

### EV-depleted fetal bovine serum preparation

Fetal bovine serum (FBS) (HYCLONE) was used as a protein source for the quantity-defined EV-protein mixture due to its high and heterogeneous protein content. FBS was heat-inactivated at 56 °C for 30 min, then ultra-centrifuged for 16 h at 150,000 × g-force to eliminate bovine-origin EVs. This depletion process is crucial for preparation of EV-protein mixture because FBS contains a large quantity of EVs. The amount of protein in the depleted FBS was determined using a Bradford protein assay, then aliquots were stored at −20 °C for later use.

### Isolation of interstitial fluid

C57BL6/j strain mice were purchased from Jackson Laboratories (Bar Harbor, ME, USA) and maintained in the specific pathogen free area in the Pohang University of Science and Technology animal facility. B16-BL6 melanoma cell line was cultured with Minimum Essential Media Alpha (MEM-α, Gibco) supplemented with 10% heat inactivated FBS and 1% antibiotics (Gibco).

One million B16-BL6 cells were injected subcutaneously into the basal body of 6 week-old C57BL6/j mice to form tumors^33^. Three weeks after this injection, tumor tissues were excised from the injected mice. Fresh tumor tissues were cut into 1–2 cm^3^ pieces and washed carefully with 5 ml phosphate-buffered saline (PBS). Each washed tumor piece was then placed in a 50-ml conical tube with 40 ml fresh PBS and incubated for 30 min at 37 °C in a humidified CO_2_ incubator. Incubated tumor pieces were carefully removed from the tube; the remaining solution was tumor interstitial fluid^34^,^35^. All procedures used in the animal experiment were approved by the Institutional Animal Care and Use Committee at POSTECH, Pohang, Republic of Korea (approval number: 2013-01-0016), and all experiments were performed in accordance with the approved guidelines and regulations.

### Isolation of EVs from interstitial fluid and validation

EVs from the tumor interstitial fluid were isolated using U/C. Briefly, collected tumor interstitial fluid was centrifuged at 800 × g-force for 10 min to remove cells, then the supernatant was centrifuged again at 3,000 × g-force for 20 min to remove cellular debris and organelles. The supernatant was diluted with PBS containing ethylenediaminetetraacetic acid (EDTA) (final concentration of 5 mM). Finally, this supernatant that was free of cells and debris was ultra-centrifuged at 100,000 × g-force for 2 h. Pelleted EVs were then resuspended with PBS, and the protein amount of EVs were quantified using a Bradford protein assay. EVs prepared from tumor interstitial fluid were validated using transmission electron microscopy (TEM), western blotting (Details in Fig. 1), and dynamic laser scattering (DLS) (Zetasizer ZS, Malvern Instrument). Morphological property of EVs isolated from interstitial fluid and culture media were compared by TEM, relative amount of the EVs’ membrane protein (CD81, CD9, and Alix) were identified by western blotting, and size of interstitial EVs were confirmed using DLS. In western blot, 2 μg of proteins from ultra-centrifuged culture media and tumor interstitial fluid was used.

### EV-protein mixture

Development and evaluation of EV isolation methods requires quantity-defined EV samples to analyze isolation efficiency by comparing pre- and post-isolation samples. This quantity-defined EV-protein mixture was prepared in 500 μl PBS with 50 μg of interstitial fluid EVs and 1,000 μg of depleted FBS proteins to simulate body fluids, which also contain varieties of proteins and cellular particles^36^,^37^,^38^. All experiments for EV isolation efficiency determination were performed using this EV-protein mixture.

### EV isolation

PEG (25–45 kDa, Sigma Aldrich) and DEX (450–650 kDa, Sigma Aldrich) were used to compose the ATPS. Various polymeric concentrations of PEG/DEX ATPS were tested to determine the optimized composition of the system. The ATPSs were prepared by dissolving the polymers directly in the EV-protein mixture at room temperature (RT) for 1 h. The solution became opaque when polymer was completely dissolved. The opaque mean that the solution formed aqueous two phase system. After vortexing, the solutions were phase-separated by centrifugation at 1,000 × g-force for 10 min at RT. In this step, EVs were isolated to bottom phase. After the phase separation, the interface between top phase and bottom phase were observed, and top phase and bottom phase could be distinguished by the interface. The top phase was composed of PEG rich solution and the bottom phase was composed of dextran rich solution. 300 μl of PEG-rich phase was carefully extracted from surface of the solution by pipette, and collected into a new tube. Solution near the phase interface was removed by pipette. Similarly, 50 μl of the remaining DEX-rich phase was also collected into a different tube for further analysis. EVs were also isolated from the EV-protein mixture using U/C and polymeric precipitation methods for comparison of isolation efficiency. Briefly, in the case of U/C, 500 μl of the EV-protein mixture was diluted with 4 ml of PBS containing EDTA (final concentration 5 mM) then ultra-centrifuged at 100,000 × g-force for 2 h. Then the supernatant was discarded and the remaining liquid around the pelleted EV was completely removed by drying for 10 min at RT. The EV pellet was resuspended in 70 μl of PBS for further analysis. This volume of PBS was the same as the volume of DEX-rich phase of system C for further analyses: 70 μl of DEX-rich phase and 430 μl of PEG-rich phase.

**Figure 5:**
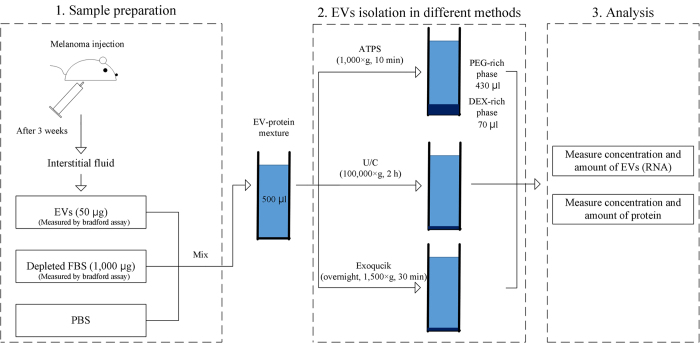
Scheme of experiments. First, 500 μl of quantity-defined EV mixture was made of PBS with 1,000 μg protein from depleted FBS and 50 μg protein from EVs previously prepared from tumor interstitial fluid. Protein amounts of depleted FBS and EVs were measured using a Bradford protein assay. Prepared EV-protein mixture was used for all subsequent isolation experiments to optimize and evaluate our ATPS method. The figure was drawn by H.S.

Polymeric EVs isolation using ExoQuick^®^ (System Biosciences Inc.) was performed according to the manufacturer’s recommendations. Briefly, 166 μl of ExoQuick^®^ solution was mixed with 500 μl of EV-protein mixture and the samples were incubated at 4 °C. After incubation, samples were centrifuged at 1500 × g-force for 30 min and the supernatant was discarded. The samples were centrifuged again at 1500 × g-force for 5 min. The supernatant was discarded and the pellet was suspended in 70 μl of PBS. Overall method was schematized in Fig. 5.

### EV and protein quantification of the pre- and post-isolation samples

The EV-protein mixtures described above contain both EVs and proteins, so conventional protein measurement cannot be used to quantify EVs; instead, this quantification was accomplished by measuring RNA amount in the samples because EVs are the only source of the RNA in the EV-protein mixture.

Pre- and post-isolation samples of 40 μl from the ATPS, U/C and polymeric methods were lysed with 0.8 ml TRI Reagent (Sigma Aldrich) for 5 min at RT. Then 0.2 ml of chloroform was added to the lysed samples and they were centrifuged at 13,500 × g-force for 10 min to separate the phases. Aqueous phase containing RNA was carefully extracted and an equal volume of isopropyl alcohol (IPA) was added to precipitate the RNA. This aqueous phase/IPA mixture was then centrifuged at 13,500 × g for 10 min to pelletize the RNA. The supernatant was discarded and the RNA pellet was washed with 75% ethanol followed, then centrifuged at 13,500 × g for 10 min. Finally, the washed RNA was dissolved in 20 μl of nuclease-free water and RNA amount was measured using a spectrophotometer (Genway, Genova) to quantify the amount of EV in the sample. The amounts of protein in the pre- and post-isolation samples were quantified using Bradford protein assays.

### Qualitative RNA profile analysis

The qualitative profiles of previously-prepared RNA samples were analyzed using Agilent RNA 6000 Nano Kit (Agilent Technology).

### Transmission electron microscopy

To visualize and examine the morphology of isolated EVs, TEM was performed; 5 μl of each isolated sample was deposited on a formavar carbon film (FCF300-cu, Electron Microscopy Science), then mixed with 7 μl of 2% uranyl acetate for 10 s to stain it. Samples were air-dried for 30 min then imaged at 60-kV acceleration voltage on a Jeol transmission electron microscope (JEM-1011, Japan).

### Western blot

Two microliters of DEX-rich phase and centrifuged EVs sample were mixed with 38 μl of distilled water and 10 μl of 5x SDS-PAGE loading buffer (250 mM Tris–HCl, 10% SDS, 0.5% bromophenol blue, 50% glycerol). The mixtures were boiled at 100 °C for 10 min, separated by SDS polyacrylamide gel electrophoresis (12% resolving gel, 120 V, 90 min) then transferred to a PVDF membrane at 390 mA, 2 h, 4 °C. The transferred PVDF membrane was treated with blocking solution (3% non-fat milk, PBS), then treated with 0.1 mg/ml CD81, CD9, or Alix primary antibody (Santa Cruz, diluted with blocking solution). Then 0.1 mg/ml HRP conjugated secondary antibody (Santa Cruz, anti-hamster IgG-HRP) in blocking solution was applied for 1 h and the presence of target protein was detected by adding chemi-luminescent substrate (Amersham Pharmacia Biotech).

### Reverse transcription-polymerase chain reaction

Reverse transcription-polymerase chain reaction (RT-PCR) was performed with 4.5 μl of the EV RNA samples prepared from DEX-rich phase and from samples obtained using U/C. RNAs were first reverse transcribed with GoScript reverse transcription kit (Promega) and amplified with GoTaq polymerase chain reaction kit (Promega) following the manufacturer’s protocol. The sequences of primers used in PCR were: Melan A forward 5′-CGCTCCTATGTCACTGCTGA- 3′ reverse 5′-GGTGATCAGGGCTCTCACAT-3′ GAPDH - forward 5′- ACC ACA GTC CAT GCC ATC AC- 3′ reverse 5′- TCCACCACCCTGTTGCTGTA-3′. The PCR program entailed thermo-cycles of 94 °C for 5 min; 40 cycles of 94, 50 and 72 °C for 30 s each; then 72 °C for 10 min. Amplified DNA samples were separated by 1% agarose gel electrophoresis with SYBR Green DNA staining agent (molecular probe). The separated bands were visualized using a BioDoc-It imaging system (UVP, Cambridge UK).

### Statistical analysis

All data are presented as means and standard deviations. Statistical analyses were performed with IBM SPSS Statistics 21 (IBM) and R-Gui software. Data were first analyzed using ANOVA and differences between pairs of means were tested using Tukey’s test. Selected pairs were tied with brackets in the figures and statistical significances of differences between pairs was designated * for p < 0.05 and as ** for p < 0.01. Two way ANOVA was performed to identify interaction between EVs and protein.

## Additional Information

**How to cite this article**: Shin, H. *et al.* High-yield isolation of extracellular vesicles using aqueous two-phase system. *Sci. Rep.* **5**, 13103; doi: 10.1038/srep13103 (2015).

## Supplementary Material

Supplementary Information
