# Supplementary material for: High-yield isolation of extracellular vesicles using aqueous two-phase system
Source: Sci Rep. 2015 Aug 14;5:13103. doi: 10.1038/srep13103 (PMC4536486; doi:10.1038/srep13103)
Supplement: Supplementary Information [file srep13103-s1.pdf]

Supplementary Information

High-yield isolation of extracellular vesicles using aqueous two-phase system

Hyunwoo Shin[a], Chungmin Han[a], Joseph M. Labuz[d], Jiyoon Kim[b], Jongmin Kim[a], Siwoo Cho[a], Yongsong Gho[c], Shuichi Takayama[de] and Jaesung Park*[ab]

Equation S1

K_s is defined as the volume fraction $\emptyset_s^b$ of solute *s* in the bottom phase divided by the volume fraction $\emptyset_s^t$ of *s* in the top phase.

$$K_s = \frac{\emptyset_s^b}{\emptyset_s^t}. \qquad (1)$$

The chemical potential μ_i^b of solute *i* in the bottom phase is expressed as

$$\mu_i^b = \left(\frac{\partial \Delta G_{mix}^b}{\partial n_i^t}\right)_{T,P,all\ n_j \neq n_i} = \mu_i^{\circ} + RTln\emptyset_i^b + (\mu_i^{ex})^b\ , \qquad (2)$$

and its chemical potential μ_i^t in the top phase is defined as

$$\mu_i^t = \left(\frac{\partial \Delta G_{mix}^t}{\partial n_i^t}\right)_{T,P,all\ n_j \neq n_i} = \mu_i^{\circ} + RTln\emptyset_i^t + (\mu_i^{ex})^t. \qquad (3)$$

where ΔG_{mix}^b and ΔG_{mix}^t are the change of Gibbs free energy in the bottom and top phase respectively, n_i^b and n_i^t are the number of solute *i* molecules in the bottom and top phase respectively, *T* is absolute temperature, *P* is pressure, μ_i° is the chemical potential of pure component *i* at system temperature, and μ_i^{ex} is the excess chemical potential of *i* in the phase.

From the phase-equilibria criteria, the system should satisfy equation (4).

$$\mu_i^t = \mu_i^b \qquad (4)$$

Subtracting equation (3) from equation (2) yields

$$\ln K_s = \ln\left(\frac{\emptyset_s^b}{\emptyset_s^t}\right) = \frac{1}{RT}[(\mu_s^{ex})^t - (\mu_s^{ex})^b]\ ; \qquad (5)$$

i.e., partition coefficient K_s is determined by μ_s^{ex} in the bottom and top phases. μ_s^{ex} is the excess entropy of mixing the phase-forming components, and the nonideal interactions between all unlike pairs present in the bottom phase (e.g., polymer-solute, polymer-solvent),

Gibbs free energy can be divided into an enthalpic term and entropic term.

$\Delta G = \Delta H - T\Delta S.$ (6)

Entropic contribution to the partition coefficient

Equation (7) is derived from equations (2) and (6), when ΔH = 0 only considering entropy.

$$\mu_i - \mu_i^{\circ} = -T\left(\frac{\partial \Delta S^c}{\partial n_i}\right)_{T,P,n_j \neq n_i} = RT\left[ln\emptyset_i - \emptyset_i + 1 - M_p \sum_{i \neq s}^{m} \frac{\emptyset_i}{M_i}\right]. \qquad (7)$$

Equation (8) can be derived from equation (7) in a manner similar to the derivation of equation (5).

$$\ln K_s = -(\emptyset_s^b - \emptyset_s^t) + M_s\left(\sum_{i \neq s}^{m} \frac{\emptyset_i^b}{M_i} - \sum_{i \neq s}^{m} \frac{\emptyset_i^t}{M_i}\right). \qquad (8)$$

$\emptyset_s^t$ and $\emptyset_s^b$ are vanishingly small ($\emptyset_s^t \approx \emptyset_s^b \approx 0$) because in the system volume of solute assumed to be much

smaller than the volume of solvent. Therefore, the equation (8) is developed to

$$\ln K_s = M_s\left(\sum_{i\neq s}^{m}\frac{\emptyset_i^b}{M_i} - \sum_{i\neq s}^{m}\frac{\emptyset_i^t}{M_i}\right) = \frac{M_s}{\rho}\left(\frac{n^b}{V^b} - \frac{n^t}{V^t}\right), \quad (9)$$

where K_s (partition coefficient) is the volume fraction of solute s in the bottom phase divided by volume fraction of solute s in the top phase, M_s is the mass of the solute, ρ is the number of lattice sites per unit volume, n^t and n^b are number of molecular of top phase and bottom phases, respectively, V^t and V^b are the volumes of top phase and bottom phase, respectively

Enthalpic contribution to the partition coefficient

If we just consider the contribution of enthalpy, equation (10, 11) is derived because entropy is zero in equation (2).

$$h_s = \left(\frac{\partial \Delta H_{mix}}{\partial n_s}\right)_{T,P,n_j\neq n_s} = -M_s \sum_{i=1(i\neq s)}^{m-1}\sum_{j=i+1(j\neq s)}^{m} \emptyset_i\emptyset_j w_{ij} + M_s \sum_{i=1(i\neq s)}^{m} \emptyset_i(1-\emptyset_s)w_{is} \quad (10)$$

$$\ln K_s = -\frac{M_s}{RT}\left[\sum_{i=1(i\neq s)}^{m}\left(\emptyset_i^b - \emptyset_i^t\right)w_{is} - \sum_{i=1(i\neq s)}^{m-1}\sum_{j=i+1(j\neq s)}^{m}\left(\emptyset_i^b\emptyset_j^b - \emptyset_i^t\emptyset_j^t\right)w_{ij}\right]. \quad (11)$$

The first term in equation (11) is a summation that is related to the energy difference induced by all binary unlike enthalpic interactions between the solute and the other components in each phase. Therefore, $\sum_{i=1(i\neq s)}^{m}\emptyset_i^b w_{is}$ represents the energy of interaction between a lattice site belonging to a protein, and the average lattice site of the bottom phase. For simplicity, $\sum_{i=1(i\neq s)}^{m}\emptyset_i^b w_{is}$ can abbreviated as w_{bs}. The second term is a summation that is related to the difference in energy of each phase due to unlike enthalpic interactions between all phase components other than the solute. Therefore, $\sum_{i=1(i\neq s)}^{m-1}\sum_{j=i+1(j\neq s)}^{m}\emptyset_i^b\emptyset_j^b w_{ij}$ represents the total enthalpy of formation of the top phase divided by the number of lattice sites in the top phase. For simplicity, $\sum_{i=1(i\neq s)}^{m-1}\sum_{j=i+1(j\neq s)}^{m}\emptyset_i^b\emptyset_j^b w_{ij}$ can abbreviated as E_b:

$$\ln K_S = -\frac{M_S}{RT}[(w_{bs} - E_b) - (w_{ts} - E_t)], \quad (12)$$

where K_s, M_s and ρ are as defined in Eq (9), R is the gas constant, T is absolute temperature, w_{ts} is energy of interaction between a lattice site belonging to a particle and an average lattice site of the top phase, w_{bs} is energy of interaction between a lattice site belonging to a particle and an average lattice site of the bottom phase, and E_t and E_b is self-energy, which is binding energy between two average lattice sites of bottom and top phases

Combining entropic (9) and enthalpic (12) contributions to the partition coefficient yields

$$\ln K_S = \frac{M_S}{\rho}\left[\left(\frac{n^b}{V^b} - \frac{n^t}{V^t}\right)\right] - \frac{M_S}{RT}[(w_{bs} - E_b) - (w_{ts} - E_t)]. \ (13)$$

References

1 Albertsson, P.-Å . Particle fractionation in liquid two-phase systems The composition of some phase systems and the behaviour of some model particles in them application to the isolation of cell walls from microorganisms. *Biochimica et biophysica acta* **27**, 378-395 (1958).

2 Walter, H. *Partitioning In Aqueous Two–Phase System: Theory, Methods, Uses, And Applications To Biotechnology*. (Elsevier, 1986).

3 Johansson, H. O., Karlstrom, G., Tjerneld, F. & Haynes, C. A. Driving forces for phase separation and partitioning in aqueous two-phase systems. *J Chromatogr B* **711**, 3-17 (1998).
